# Draft genome assemblies of three *Klebsiella grimontii* strains isolated from catheterized urine samples from the same male participant over the course of 6 months

**DOI:** 10.1128/mra.00506-24

**Published:** 2024-07-08

**Authors:** Helen Appleberry, Warwick Price, Lorenzo Roque, Evelyn Umana, Alan J. Wolfe, Catherine Putonti, Alex Kula

**Affiliations:** 1 Department of Biology, Loyola University Chicago, Chicago, Illinois, USA; 2 Bioinformatics Program, Loyola University Chicago, Chicago, Illinois, USA; 3 Department of Microbiology and Immunology, Loyola University Chicago, Maywood, Illinois, USA; DOE Joint Genome Institute, Berkeley, California, USA

**Keywords:** *Klebsiella grimontii*, urinary tract, urobiome, urinary tract infection

## Abstract

*Klebsiella grimontii* is a newly identified species within the *Klebsiella oxytoca* complex. Here, we present the draft genome sequence of three *K. grimontii* strains that were isolated from catheterized urine samples collected from a participant in a longitudinal study over ~6 months.

## ANNOUNCEMENT

*Klebsiella grimontii* is a newly characterized species within the *Klebsiella oxytoca* complex (1, 2). *K. oxytoca* is a common cause of urinary tract infections (UTI) (2). *K. grimontii* strains have been isolated from wound infections, respiratory tract infections, and antibiotic-associated hemorrhagic colitis (1) as well as urine samples (3). As part of a prior institutional review board (IRB)-approved study characterizing the urobiome of geriatric males with chronic indwelling urinary catheters, *K. grimontii* was isolated from a participant across three urine samples collected during this longitudinal study. These three *K. grimontii*-containing collections span ~6 months. Here we present the genome assemblies of *K. grimontii* UMB12529, UMB12659, and UMB12885.

Urine samples collected (IRB approval nos. LU212677 and LU217801) were processed using the expanded quantitative urine culture (EQUC) method (4). Each isolate was identified as *K. oxytoca via* matrix-assisted laser desorption ionization-time of flight mass spectrometer (MALDI-TOF MS; Bruker Daltonics, Billerica, MA), as previously described (5), and preserved at −80°C in the Loyola Urinary Education and Research Collaborative (LUEREC) collection. Samples were obtained from this collection and streaked onto tryptone soy agar (TSA) plates and incubated at 35°C with 5% CO_2_ for 24 h. Individual colonies were then grown in liquid tryptone soy medium under the same culture conditions. These liquid cultures were used for DNA extraction with Qiagen’s DNeasy Blood and Tissue kit, following the manufacturer’s Gram-positive organism protocol. Library preparation and sequencing were performed by SeqCoast (Portsmouth, NH USA). DNA libraries were constructed using the Illumina tagmentation kit and unique dual indexes and sequenced on the Illumina NextSeq2000 platform using a 300-cycle flow cell kit to produce 2 × 150 bp paired reads. Reads were processed with the Bacterial and Viral Bioinformatics Resource Center (BV-BRC) v3.35.5 webtool (6) as follows. Reads were first trimmed using Trim Galore v0.6.5 (https://github.com/FelixKrueger/TrimGalore) and assembled using Unicycler v0.4.8 (7). The draft assembly was polished with Pilon v1.23 (8), which corrects bases, fixes misassemblies, and fills gaps. Genome coverage, completeness, and contamination were computed by BV-BRC. Genome assemblies were annotated by the NCBI Prokaryotic Genome Annotation Pipeline (PGAP) v6.7 (9). BV-BRC’s Bacterial Genome Tree tool was used to compare the genomes (parameter 1000 genes); the resulting Newick-format tree was visualized using iTOL v6 (10). Variant analysis was conducted using BV-BRC by performing all pairwise comparisons of raw reads to genome assemblies; only high-quality variants were recorded. Unless otherwise specified, default parameters were used for all software.

**Fig 1:**
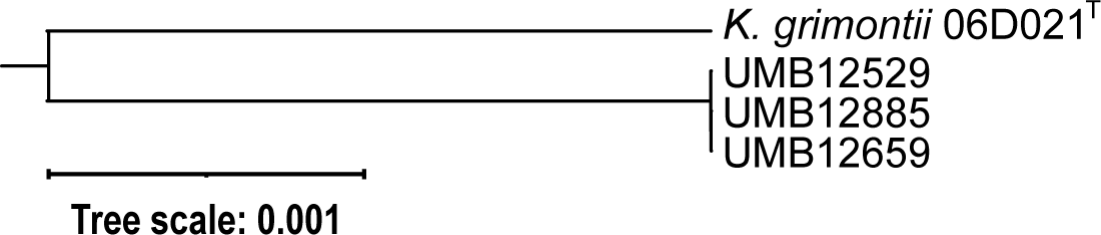
Phylogenetic tree based upon 1,000 genes shared between the three *K. grimontii* strains presented here and the type strain for the species.

Genome assembly statistics are shown in Table 1. The *K. grimontii* UMB12529, UMB12659, and UMB12885 genome assemblies are nearly identical and distinct from *K. grimontii* strain 06D021^T^ (Accession no. FZTC00000000) (Fig. 1). Variant analysis found that 59–75 SNPs distinguish the three genomes. The high sequence similarity suggests that the same strain of *K. grimontii* has persisted in the participant’s urobiome. Given the recent characterization of new species within the *K. oxytoca* complex, including *K. grimontii*, and the known shortcomings of MALDI-TOF to distinguish between these species (3), future studies are needed to disambiguate between *K. oxytoca* complex species and urinary tract symptoms.

**TABLE 1:** Genome assembly and strain information about the three *K. grimontii* strains

Strain	UMB12529	UMB12659	UMB12885
SRA accession No.	SRR28710908	SRR28710907	SRR28710911
Assembly accession No.	JBCGEM000000000	JBCGEK000000000	JBCGEL000000000
No. raw reads	2,721,256	1,761,298	3,218,480
Assembly length (bp)	6,169,055	6,150,323	6,148,987
G + C (%)	55.58	55.59	55.59
No. contigs	132	129	133
Contigs N50 (bp)	185,793	211,986	186,216
Coverage (x)	60.16	39.56	71.21
Completeness (%)	100	100	100
Contamination (%)	0.6	0.4	0.4
Collection date	May 17, 2021	July 22, 2021	November 5, 2021
Symptom status	Asymptomatic	Asymptomatic	UTI

## Data Availability

Table 1 lists the SRA accession numbers and assembly accession numbers for the three strains.
